# Cross-Cultural adaptation of the General Functioning Scale of the Family

**DOI:** 10.1590/S1518-8787.2016050005832

**Published:** 2016-06-17

**Authors:** Thiago Pires, Simone Gonçalves de Assis, Joviana Quintes Avanci, Renata Pires Pesce

**Affiliations:** Departamento de Estudos sobre Violência e Saúde Jorge Careli. Escola Nacional de Saúde Pública Sergio Arouca. Fundação Oswaldo Cruz. Rio de Janeiro, RJ, Brasil

**Keywords:** Family Relations, Cross-Cultural Comparison, Questionnaires, Translations, Reproducibility of Results, Validation Studies

## Abstract

**OBJECTIVE:**

To describe the process of cross-cultural adaptation of the General Functioning Scale of the Family, a subscale of the McMaster Family Assessment Device, for the Brazilian population.

**METHODS:**

The General Functioning Scale of the Family was translated into Portuguese and administered to 500 guardians of children in the second grade of elementary school in public schools of Sao Gonçalo, Rio de Janeiro, Southeastern Brazil. The types of equivalences investigated were: conceptual and of items, semantic, operational, and measurement. The study involved discussions with experts, translations and back-translations of the instrument, and psychometric assessment. Reliability and validity studies were carried out by internal consistency testing (Cronbach’s alpha), Guttman split-half correlation model, Pearson correlation coefficient, and confirmatory factor analysis. Associations between General Functioning of the Family and variables theoretically associated with the theme (father’s or mother’s drunkenness and violence between parents) were estimated by odds ratio.

**RESULTS:**

Semantic equivalence was between 90.0% and 100%. Cronbach’s alpha ranged from 0.79 to 0.81, indicating good internal consistency of the instrument. Pearson correlation coefficient ranged between 0.303 and 0.549. Statistical association was found between the general functioning of the family score and the theoretically related variables, as well as good fit quality of the confirmatory analysis model.

**CONCLUSIONS:**

The results indicate the feasibility of administering the instrument to the Brazilian population, as it is easy to understand and a good measurement of the construct of interest.

## RESUMO

**OBJETIVO:**

Descrever o processo de adaptação transcultural da escala de Funcionamento Geral da Família, subescala da *McMaster Family Assessment Device*, para a população brasileira.

**MÉTODOS:**

A escala de Funcionamento Geral da Família, original no idioma inglês, foi traduzida para o português e aplicada a 500 responsáveis de crianças do segundo ano do ensino fundamental de escolas públicas do município de São Gonçalo, Rio de Janeiro. Os tipos de equivalências investigados foram: conceitual e de itens, semântica, operacional, e mensuração. O estudo envolveu discussão com especialistas, traduções e retraduções do instrumento e avaliação psicométrica. Foi realizado estudo de confiabilidade e validade, por testagem da consistência interna (alpha de Cronbach), da correlação pelo método de *split-half* de Guttman e do coeficiente de correlação de Pearson, e por análise fatorial confirmatória. Associações entre Funcionamento Geral da Família e variáveis teoricamente associadas ao tema (embriaguez do pai ou da mãe e violência entre os pais) foram analisadas por estimação de razão de chance.

**RESULTADOS:**

A equivalência semântica encontrada foi entre 90,0% e 100%. O alfa de Cronbach variou de 0,79 a 0,81, indicando boa consistência interna do instrumento. O coeficiente de correlação de Pearson oscilou entre 0,303 e 0,549. Encontrou-se associação estatística entre o escore de funcionamento geral da família e as variáveis teoricamente relacionadas e constatou-se boa qualidade dos ajustes do modelo de análise confirmatória.

**CONCLUSÕES:**

Os resultados obtidos indicam a aplicabilidade do instrumento na população brasileira, que se mostra de fácil compreensão e boa aferição do constructo de interesse.

## INTRODUCTION

There is no single concept or model defining “family functioning”. The issue is addressed in multiple ways in the literature from different perspectives and under different names, among which are family functioning, family dynamics, family satisfaction, parental practice and styles, and family support. The complexity of the field is compounded by the variety of theoretical models and evaluation measures, and the lack of consensus on the definitions of dysfunctional and healthy family^19^. However, theorists agree that the family cannot be understood merely by the individual study of behaviors or relationships; this knowledge must be evaluated together with the family environment created by the synergistic interaction among its members.

The literature features quantitative, qualitative^2^ and mixed^22^ approaches to understanding the functioning of a family. The quantitative tools include the following, each one based on specific theoretical assumptions, reflecting the diversity predominant in the area: a) Family Assessment Device^7^, which describes the structure, the organization of the family unit, and the relationship patterns among family members; b) Family Environment Scale^12^, an instrument based on socio-ecological and psychological theory, and the family systems theory, which evaluates social environment characteristics of all types of families; c) Family Assessment Measure^18^, based on the systems model in the marital and family environment, assesses family cohesion and adaptability and the difference between ideal and actual family functioning; d) Family Adaptability Cohesion Evaluation Scale III^14^, which emphasizes family dynamics and the interaction between individual and family processes; e) Family Perceptions Scale^25^, which is based on knowledge of different subjectivities, creating multiple family realities. The main aspects raised in these family functioning scales are: family roles, values and norms, communication, affective involvement, and problem-solving^19^.

One of the most commonly used scales is the Family Assessment Device, a self-reporting tool based on the McMaster^7^ family functioning model, designed in the United States. Aarons et al.^1^ report its extensive use in recent decades in different continents: Asia, with studies in China and Japan; Europe, with research in Italy, the Netherlands, Hungary and England; America, with research in Canada; besides Oceania, with studies in Australia. The research covers various ethnic groups, including Japanese, Greeks living in Australia, Hispanic Americans and Hawaiian Americans.

The theoretical model underlying the Family Assessment Device evaluates six family functioning dimensions (41 items): problem-solving (the family’s ability to solve problems to establish effective family functioning); communication (how information is orally transmitted in the family); family roles (behavior patterns of individuals in the functioning of the family); affective responsiveness (family’s ability to respond to affective experiences with appropriate feelings); affective involvement (frequency and level of interest and investment among family members); and behavior control (pattern adopted by the family to respond to physically hazardous situations involving the expression of psycho-biological needs and socializing behaviors). The device also enables assessment of the general functioning of the family (GFF) via 12 items^7^. The full version of the scale comprises a total of 53 items.

A systematic review^19^ of the literature published between January 1990 and July 2009 on the assessment of family functioning in psychiatry, carried out in the MEDLINE, PubMed and PsycInfo databases, showed that 13 of the 20 international studies found used the Family Assessment Device. In this sense, this article aims to describe the cross-cultural adaptation process of the GFF scale for use with the Brazilian population, according to the proposal operationalized by Reichenheim and Moraes^15^.

## METHODS

The sampling plan is based on registers of public schools, classes and average number of students per class, provided by the Municipal Department of Education of Sao Gonçalo, RJ, Southeastern Brazil, for the year of 2005 (universe of 6,589 students in the second grade of elementary school). The sample design uses three selection stages (schools, second grade classes and students), which included 25 schools randomly selected by systematic sampling with probability proportional to size (PPS). Two classes per school and 10 students per class were randomly selected, totaling 500 students in the respective year of elementary school. Approximately 35.0% of the originally selected students were replaced by the next on the list, mainly due to failures in the attendance diary.

The first version of the Family Assessment Device has 240 items (40 for each one of the six dimensions). To reduce this version, the authors adopted some procedures to define a scale to represent general functioning, consisting of the items that best correlate with the six dimensions. Twelve items were selected: one from problem-solving, four from communication, two from family roles, one from affective responsiveness, three from affective involvement, and one from control behavior. The following criteria were used to reduce the items in each one of the six dimensions: relevance of the theme in the dimension; set of items with high correlation and high internal consistency; and items more correlated with their own dimension than with the others. At this stage, the items that made up the six scales were preserved in case they contributed to raise Cronbach’s alpha (above 0.7) and there were no more items capable of increasing alpha or improving the correlation of the scale with the others. The high correlation of the items of the general functioning scale with the six family dimensions investigated support the worldwide use of this smaller scale.

The GFF, a subscale of the General Functioning Scale of the McMaster Family Assessment Device^7^, includes 12 questions: planning family activities is difficult because we misunderstand each other; in times of crisis, we can turn to each other for support; we cannot talk to each other about the sadness we feel; individuals are accepted for what they are; we avoid discussing our fears or concerns; we express feelings to each other; there are lots of bad feelings in our family; we feel accepted for what we are; making decisions is a problem for our family; we are able to make decisions about how to solve problems; we don’t get along well together; we can confide in each other. The response options range from “strongly agree” to “strongly disagree” (1-5 points), with higher values meaning better GFF (Table 1). The same number of items and response options were preserved for use with the children of Sao Gonçalo. The instrument was addressed to the children’s guardians, in most cases the mother, and administered by trained psychologists and social workers who relied on a manual to guide the questionnaire.

**Table 1:** General Functioning Scale of the Family used in the study.

The statements below refer to the functioning of your family. Indicate whether you strongly agree; agree; neither agree nor disagree; disagree; or strongly disagree	
1. Planning family activities is difficult because you can misunderstand each other	
1. Strongly agree	4. Disagree
2. Agree	5. Strongly disagree
3. Neither agree nor disagree	9. Does not know
2. In times of crisis you can turn to each other for support	
1. Strongly agree	4. Disagree
2. Agree	5. Strongly disagree
3. Neither agree nor disagree	9. Does not know
3. You cannot talk among yourselves about the sadness you feel	
1. Strongly agree	4. Disagree
2. Agree	5. Strongly disagree
3. Neither agree nor disagree	9. Does not know
4. Individuals in the family are accepted for what they are	
1. Strongly agree	4. Disagree
2. Agree	5. Strongly disagree
3. Neither agree nor disagree	9. Does not know
5. You avoid discussing your fears or concerns	
1. Strongly agree	4. Disagree
2. Agree	5. Strongly disagree
3. Neither agree nor disagree	9. Does not know
6. You express feelings to each other	
1. Strongly agree	4. Disagree
2. Agree	5. Strongly disagree
3. Neither agree nor disagree	9. Does not know
7. There are lots of bad feelings in your family	
1. Strongly agree	4. Disagree
2. Agree	5. Strongly disagree
3. Neither agree nor disagree	9. Does not know
8. You feel accepted for what you are	
1. Strongly agree	4. Disagree
2. Agree	5. Strongly disagree
3. Neither agree nor disagree	9. Does not know
9. Making decisions is a problem for your family	
1. Strongly agree	4. Disagree
2. Agree	5. Strongly disagree
3. Neither agree nor disagree	9. Does not know
10. You are able to make decisions about how to solve problems	
1. Strongly agree	4. Disagree
2. Agree	5. Strongly disagree
3. Neither agree nor disagree	9. Does not know
11. You don’t get along well together.	
1. Strongly agree	4. Disagree
2. Agree	5. Strongly disagree
3. Neither agree nor disagree	9. Does not know
12. You can confide in each other	
1. Strongly agree	4. Disagree
2. Agree	5. Strongly disagree
3. Neither agree nor disagree	9. Does not know

The adaptation followed the method proposed by Herdman et al.^9^ and was systematized by Reichenheim and Moraes^15^. The following steps feature in this article: a) conceptual and items equivalence: this evaluates whether the items that comprise the studied scale estimate the same areas and are relevant in the original culture and in the Brazilian culture. It involved literature review and discussion of the concepts with experts; b) semantic equivalence: this consists of translations and back-translations of the original instrument, not only preserving the meaning of words between two different languages, but also seeking to achieve the same semantic-emotional effect between different cultures. Initially, two translations from English to Portuguese were done independently by professionals with knowledge of English. Next, two back-translations were done, also independently, by two other professionals familiar with the English language and culture. A fifth professional assessed the agreement between the original items and the two back-translations. This evaluation was done blindly, making it impossible to distinguish between the original item and the one that was translated and back-translated. Two categories of semantic equivalence between the items were evaluated by this professional: referential meaning (agreement in terms of literal translation between the original item and the same one back-translated, ranging from 0% to 100%), and general meaning (broader agreement in terms of articulation of ideas and impact between an original item and its back-translation, ranging from unaltered to slightly altered, significantly altered or completely altered). The professional in charge of this step made comments and gave suggestions to match the back-translated items to the original items as closely as possible and adapt them to the target population. These comments were discussed by the authors of this article, who decided on the Portuguese version. Lastly, the final version of the scale was administered to the guardians of the children composing the sample of this study, seeking to observe whether the scale was adequately understood by the target population, and to evaluate its psychometric properties; c) operational equivalence: this assesses the relevance and adequacy of the format of questions and instructions, the setting and mode of administration, and the categorization mode; d) measurement equivalence: this refers to the psychometric study, assessed by reliability and validity measures.

The internal consistency of the scale was measured by: a) Cronbach’s alpha coefficient, which evaluates to what extent the items are homogeneous when measuring the same construct (estimated overall and by deleting an item); b) Guttman split-half method; c) correlation of each item of the scale with the full scale, using the Pearson correlation coefficient. These measures support the understanding of the scale’s conceptual equivalence and, therefore, of the validity aspects.

Construct validity was assessed by correlation with concepts that are theoretically relevant to the theme. These are: father or mother who drinks to a state of intoxication^3^ ^,^ ^6^ ^,^ ^23^ ^,^ ^24^ and father’s violence against the mother or vice versa. The presence of violence was measured by the Conflict Tactics Scale (CTS-1), developed by Straus^21^. This analyzed severe physical violence committed by the father or mother to each other: punching, kicking, attempting to hit with objects, beating, threatening or actually using firearms or knives. A positive item indicates the presence of the father’s violence against the mother or of the mother’s against the father.

An associations analysis was carried out between GFF and the variables by odds ratio (OR) estimation, calculated from the coefficients of the logistic regression models, considering the complex sample design in calculating standard errors and the sample weights in correcting one-off estimates. The GFF scale was categorized as having the median as a cutoff point.

Confirmatory factor analysis is the last step in investigating measurement equivalence. To examine the latent structure of GFF, confirmatory factor analysis was applied to verify the number of underlying dimensions of the instrument (factors) and how the item-factor relationship (factor loadings) is manifested. This technique can provide important evidence on the instrument’s convergent and discriminant validity. Expressiveness of factor loadings, residual variances and modification indexes were analyzed. The estimation method applied was Mean- and Variance-adjusted Weighted Least Square (WLSMV)^4^. The quality measure of the model fit was Root Mean Square Error of Approximation (RMSEA), with < 0.05 considered as a good fit value. Good results for the Comparative Fit Index (CFI) and the Tucker-Lewis Index (TLI), on the other hand, are within the range of > 0.90. Modification indexes with values greater than 10, which indicate that a particular factor loading or residual correlation must be estimated, were investigated. The software program used to perform the analysis was R 2.13.0.

The study was approved by the Research Ethics Committee of the National School of Public Health in 2005 (Process 0051.0.031.000-04). The school and the students’ parents signed an informed consent form.

## RESULTS

Most of the children taking part in the study were male (51.6%), between six and eight years old (74.6%), were *pardos* (54.0%), lived with both their father and mother (52.8%), and belonged to the C, D or E (95.0%) socioeconomic groups^a^, of lower purchasing power.

The literature review on the topic and the discussion among experts in the area indicated that the GFF scale was relevant to our culture. The originally proposed unidimensionality was considered as belonging to a construct that effectively evaluates the functioning of a family. The option was made to maintain the 12 items, trying to preserve their original meanings (Table 2).

**Table 2:** Comparison between the items of the original General Functioning Scale of the Family and the final Portuguese version.

Original (English)	Final version (Portuguese)*
Planning family activities is difficult because we can misunderstand each other.	*É difícil planejar atividades familiares porque vocês se desentendem entre si. (1)*
In times of crisis we can turn to each other for support.	*Em tempos de crise, vocês podem buscar ajuda uns nos outros. (2)*
We cannot talk to each other about sadness we feel.	*Vocês não podem conversar entre vocês sobre a tristeza que sentem. (3)*
Individuals in the family are accepted for what they are.	*Cada pessoa da família é aceita pelo que ela é. (4)*
We avoid discussing our fears or concerns.	*Vocês evitam discutir seus medos ou preocupações. (5)*
We express feelings to each other.	*Vocês mostram sentimentos uns com os outros. (6)*
There are lots of bad feelings in our family.	*Existem muitos sentimentos ruins na sua família. (7)*
We feel accepted for what we are.	*Vocês se sentem aceitos pelo que são. (8)*
Making decisions is a problem for our family.	*Tomar decisões é um problema para a sua família. (9)*
We are able to make decisions about how to solve problems.	*Vocês são capazes de tomar decisões sobre como resolver os problemas. (10)*
We don’t get along well together.	*Vocês não se dão bem juntos. (11)*
We can confide in each other.	*Vocês confiam uns nos outros. (12)*

* In the research, due to the need to administer the scale in interview form, i.e., to read the questions to adults, the GFF scale was adapted to second person plural. In case of self-reporting, first person plural can be used.

The Portuguese version was also checked for items that should be replaced for not having the same connotation of the original term. All items were kept, which also relates to the study of equivalences presented below.

As to the assessment of semantic equivalence, the referential meaning showed good results, with almost all items obtaining an agreement level in this criterion ranging from 90.0% to 100% in at least one of the back-translations (most commonly in both back-translations). Only one item caused problems in both back-translations: “We cannot talk to each other about the sadness we feel”. In the general meaning analysis, most of the items were considered unaltered or slightly altered in relation to the original instrument in both back-translations. The same item that presented problems in the referential meaning was considered significantly altered in its general meaning. In this sense, the researchers chose to use the following translation: “*vocês não podem conversar entre vocês sobre a tristeza que sentem*” (You cannot talk among yourselves about the sadness you feel). Table 2 shows the GFF scale in English and Portuguese.

Cronbach’s alpha for the total scale was 0.814 and Guttman split-half correlation presented a similar coefficient (0.800), indicating good internal consistency of the instrument.

As for the correlation of the items following the exclusion of each one in relation to the full scale, Cronbach’s alpha ranged from 0.793 to 0.817. Therefore, no deletion of a single item of the instrument caused a considerable increase of the studied coefficients, suggesting that all items are directly proportional to the scale score. In addition, the Pearson correlation coefficient (item-scale) ranged between 0.303 and 0.549.

The construct validity study showed that families in which mothers or fathers drink to a state of intoxication have a higher chance of achieving scores above the median, indicating poor family functioning, and families in which mothers or fathers committed severe physical violence against each other have a higher probability of experiencing worse family functioning (Table 3).

**Table 3:** Odds ratio and 95% confidence interval between selected variables and the GFF scale, with the GFF score categorized by the median.

Variable	OR	95%CI
Mother drinks to the point of intoxication	1.981	1.014–3.873
Father drinks to the point of intoxication	2.066	1.244–3.432
Severe physical violence of mother against father	2.899	1.348–6.235
Severe physical violence of father against mother	2.929	1.369–6.265

GFF: general functioning of the family

Confirmatory factor analysis was applied according to the theoretical structure defined in Figure A. Fit quality indexes were reasonable. RMSEA of 0.101 above the tolerable limit indicates good fit. CFI and TLI showed better results with values equal to 0.922 and 0.905, respectively, and within the good fit range. The lowest standardized factor loading was found in item 5 (0.324), explained by an indicator variability of 10.4%, and in item 9 (0.558) (explained variability of 31.1%). The highest value is displayed in item 8, with a 0.712 loading, which explains 51.5% of variability. The assessed convergent validity was good, with a value of 0.878.

**Figure:**
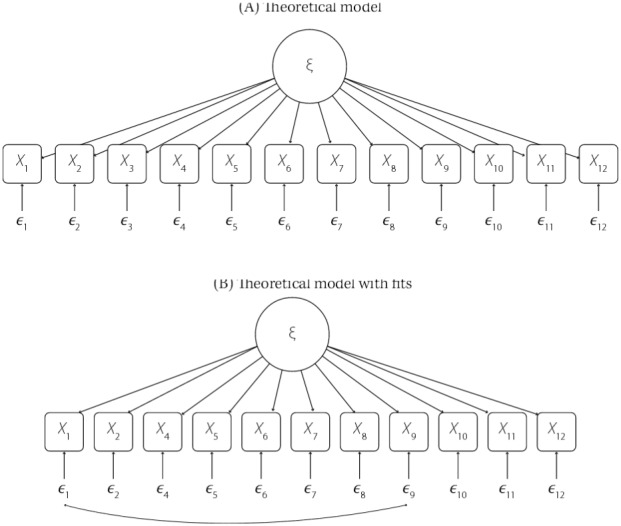
Theoretical factor structure of the General Functioning Scale of the Family.

Evaluating the modification indexes (mi) with values above 10, the following covariances are suggested: items 3 and 5 (mi = 35.889) items 3 and 12 (mi = 12.169), items 4 and 8 (mi = 11.804), items 1 and 3 (mi = 10,630), and items 2 and 6 (mi = 10,543). These autocorrelations may suggest the overlap of some items, especially item 3, “We cannot talk to each other about the sadness we feel,” which is correlated with three other items: 1, “Planning family activities is difficult because we misunderstand each other;” 5, “We avoid discussing our fears or concerns;” and 12, “We confide in each other”.

After the analysis of the modification indexes suggested restructuring the model, item 3 was removed and the residual autocorrelation between items 1, “Planning family activities is difficult because we misunderstand each other” and 9, “Making decisions is a problem in our family” (Figure, B) was included. The RMSEA for this model was 0.079, with an upper limit of 0.092 for the 90% confidence interval. The other CFI and TLI indicators were 0.958 and 0.946, respectively. Table 4 summarizes the indexes of the two models tested, in which the theoretical model with fits started showing better results.

**Table 4:** Fit quality indexes of structures tested by CFA.

Structures	Chisq	Df	CFI	TLI	RMSEA (90%CI)
Theoretical model	298.045	54	0.922	0.905	0.101 (0.090–0.112)
Theoretical model with fits*	162.542	43	0.958	0.946	0.079 (0.066–0.092)

CFA: confirmatory factor analysis; Chisq: Chi-square; Df: degree of freedom; CFI: comparative fit index; TLI: Tuker-Lewis index; RMSEA: root mean square error for approximation

* Without item 3 and the correlation estimate between items 1 and 9.

Table 5 features the standardized factor loadings of the final model and the autocorrelation of the residues between indicators 1 and 9. The lowest coefficient is of item 5, “We avoid discussing our fears or concerns” (0.269), and the highest is of item 12, “We confide in each other” (0.735). The autocorrelation between the indicators was 0.220.

**Table 5:** Estimated coefficients of the confirmatory factor analysis final model. Factor loadings and autocorrelation of residues of items 1 and 9.

Variables	Coefficients	Standard Error
ξ → x_1_	0.561	-
ξ → x_2_	0.642	0.051
ξ → x_4_	0.649	0.049
ξ → x_5_	0.269	0.044
ξ → x_6_	0.599	0.049
ξ → x_7_	0.652	0.047
ξ → x_8_	0.654	0.050
ξ → x_9_	0.507	0.044
ξ → x_10_	0.678	0.049
ξ → x_11_	0.701	0.050
ξ → x_12_	0.735	0.050
x_1_ → x_9_	0.220	0.049

ξ → x_i_: factor loadings.

x_i_ → x_j_: autocorrelation of residues.

## DISCUSSION

The items adapted from the original scale into Portuguese were mostly slightly altered or unaltered. They proved to be easily understood by the guardians who were interviewed, most of whom had a low level of schooling. Unlike the more frequent self-reporting use of the scale in international contexts, the instrument was verbally administered by researchers to mothers or guardians of children studying in the public school investigated. These findings contribute to the functional equivalence of the scale.

The adequate grasp of the semantic equivalence between the proposed and original versions is conspicuous, with only one item requiring more attention and care in adjusting to the Portuguese language and Brazilian culture. However, this item was kept due to the relevance of the theme and scale comparability with international studies.

The data on measurement equivalence presented in this article indicate good construct validity and reliability of the instrument adapted to the Brazilian version. The correlations of each item and the full scale were statistically significant; however, variation was broad (0.303 and 0.549). Internal consistency was satisfactory and the removal of any individual item did not cause significant change in the index, enhancing the adequacy of its validity.

The associations of the constructs theoretically related to the GFF scale inclined towards the assumed results^8^, i.e., worse functioning among those families in which the mother or father drink to a state of inebriation^24^ or engage in interpersonal violence against each other^6^.

Schek^17^ adapted the general functioning scale of the family to another cultural context, with a sample of 4,000 Chinese adolescents. The author obtained significant results similar to those investigated here: temporal stability and good internal consistency, good construct and concurrent validity, and the ability to discriminate between clinical and nonclinical groups. The author points out that the significant correlation of the GFF scale with other family functioning measures is a relevant finding, especially since the GFF is a fairly reduced scale compared to the others. This result is at odds with Morris^13^ and Aarons et al.^1^, who concluded that the scale might have weaker results in non-Western populations.

Other studies report GFF scale measurement equivalence data. Georgiadis et al.^8^ found internal consistency assessed by alpha 0.89 and Zubrick et al.^b^ found alpha 0.88, similar to those found here, around 0.80.

The theoretical model based on a few fits showed better results, with the exclusion of item 3, “We cannot talk to each other about the sadness we feel,” whose residue correlated with that of other three items. This may indicate that this item represents the same idea of other items, therefore being redundant in this instrument. In the analysis, items 2 and 9 were maintained, despite the correlation. Operationally, this option would allow for decision-making in the analysis phase, for example, by calculating the score of only one of the answers – the one considered most serious, since the information gathered may be redundant. Both changes – deletion of item 3 and choice of the most serious response between the items 2 and 9 – should be investigated in other samples before a final decision in favor of its exclusion and of reducing the scale in the national context.

As to the limitations of this study, there was no assessment of cutoffs points to distinguish between “pathological” and “healthy” family functioning in the Brazilian population. Some international studies use different cutoff points above the average to define families with functioning problems: 1.75, 2.00 and 2.17^5^ ^,^ ^8^ ^,^ ^11^; however, there is no consensus. Moreover, due to the fact that the survey was carried out with a representative sample from the public school system of Sao Gonçalo, RJ, the scale may show a different behavior in other Brazilian socioeconomic strata or cultural contexts. In addition, the results are restricted to information given by Brazilian adults (guardians of the children and adolescents in the survey), and lack information on the understanding of the scale by adolescents. The viewpoint of adolescents is often more negative than that of adult family members^16^.

## Footnotes

a Associação Brasileira de Institutos de Pesquisa de Mercado. Caracterização socioeconômica. São Paulo (SP): ABIPEME; 2008.

b Zubrick SR, Silburn SR, Garton AF, Dalby R, Carlton J, Shepherd C, et al. Western Australian Child Health Survey: developing health and well-being in the Nineties. Perth: Institute for Child Health Research; 1995.
